# Proliferation versus regeneration: the good, the bad and the ugly

**DOI:** 10.3389/fphys.2014.00010

**Published:** 2014-01-28

**Authors:** George I. Lambrou, Eumorphia Remboutsika

**Affiliations:** ^1^Choremeio Research Laboratory, First Department of Pediatrics, University of AthensAthens, Greece; ^2^Stem Cell Biology Laboratory, Biomedical Sciences Research Centre “Alexander Fleming”Vari-Attica, Greece

**Keywords:** stemness, stem cells, cancer, proliferation, regeneration

## A brief introduction

Adult stem cells represent quiescent cells that once prompted, they proliferate with the potential to differentiate into a range of progeny providing regenerative medicine with novel tools for cure and rehabilitation. However, the risk that these proliferating stem cells could easily become a tumor cell sets barriers for their use. But, how are stem cells involved in tumor formation? The answer is that they possess a common mechanism: their ability for proliferation, differentiation, and of self-renewing beyond the body's capabilities. This exact trait is that makes stem cells so promisable and terrifying at the same moment, leading to *Erebus*. Tumors are interpreted in two distinct ways. Either as a disease, where the cause is interpreted as a cellular/genomic malfunction or as a reaction where the cause is interpreted as a natural consequence of evolutionary origin. As a result, treatment modalities could depend on this initial interpretation.

Generally though, tumorigenesis is described as a normal cell initially being transformed to a tumor cell that starts to proliferate. This cell could be a differentiated somatic cell or an adult stem cell that could develop into a cancer stem cell (CSC). It is crucial that a diagnosis of the tumor to take place before any therapeutic intervention. Most of the times therapies involve surgical or chemical elimination of the tumor. Thus at that point, the tissue need for reconstruction would most probably implicate new stem cells that possess the ability to recognize the site of defect, locate it and initiate the reconstruction, which involves regeneration.

## The stem cell theories of carcinogenesis

The link between stem cells and tumorigenesis remains quite elusive. Tumors could stem from CSCs (Oishi and Wang, 2011), as evidence suggests for different tumors types, such as glioblastoma (Singh et al., 2003, 2004) breast cancer (Jain and Alahari, 2011), ovarian cancer (Curley et al., 2011) and gastrointestinal cancer (Davies et al., 2011; Skoudy et al., 2011; Zheng et al., 2011). Furthermore, there is a strong debate on the existence or not of a rare CSC population in childhood acute lymphoblastic leukemia (ALL). An interesting report by Greaves (2010) highlights that there are three main attributions to cancer propagation: (a) a fixed and hierarchically positioned subset of stem cells resembling normal hematopoietic stem cells, (b) a non-deterministic or stochastic process with plasticity of “stemness” and (c) the activity of a genetically dominant sub-clone (Greaves, 2010). Above recent findings suggest also a definite role of stem cells in carcinogenesis.

## Cancer: the wound that never heals

Tumors have been considered as wounds that never heal, while the connections between cancer, inflammation and local tissue repair have been reported extensively. These are very interesting topics, which brings up the notion that different phenotypes of cellular pathology could emerge from similar gene regulatory mechanisms. An interesting concept is presented by Riss et al. (2006) who proposes that tissue regeneration and tumorigenesis are probably two relatives that differ in some minor details. Both phenomena are very complex, they do however share some common characteristics. The application of the term “wound” was given to cancer by Dvorak (1986), highlighting the fact that both wound healing and tumor development require a microenvironment and a support of stromal cells in order to occur.

## The microenvironment

Last but not least, an interesting question arises. What does the microenvironment, or in other words “the niche”, have to contribute for cell reconstruction? An interplay between CSCs and microenvironment has been observed in colon cancer, where the pluripotency of CSCs is maintained at the base of colon crypts influenced by fibroblast, endothelium and inflammatory cells, cytokines and growth factors secreted by these cells (in particular HGF) and by that means ultimately the balance between self-renewal and differentiation of the staminal population is regulated (Adegboyega et al., 2002; De Sousa et al., 2011; Catalano et al., 2013). Epithelial-Mesenchymal Transition (EMT)-inducing factors released by the surrounding tumor microenvironment can affect the invasive phenotype in the initiation of epithelial malignancies (Le et al., 2008). Key regulators of this process are TGFA, through the activation of Twist, SLUG, ZEB2, and PI3K/Akt, which increase the mTOR kinase expression, Shh and Wnt (Gulhati et al., 2011). As feeding becomes an urgent requirement to support the rapid growth of the tumor, microenvironmental stimuli facilitate CSCs vasculature crosstalk. Indeed, tumor microenvironment orchestrates a vascular niche formation, determining the fate of CSCs (Calabrese et al., 2007). Numerous reports have highlighted the “*rail tracks*” metaphor and the crucial role of the microenvironment in the development and maintenance of tumors as well as healthy tissues. For example, such a study mentioned the existence of variant subpopulations within the same ovarian tumor emerging under the influence of different microenvironments (Abelson et al., 2013). An interesting debate is focused on the role of mesenchymal stem cells, as they can manifest a dual mechanism of action, that is tumor-inducing as well as tumor suppressing, linking the role of the microenvironment to tumor progression (Yagi and Kitagawa, 2013). Stem cells, as neural stem cells, employ intrinsic factors together with extrinsic determinants to acquire flexibility in their response to their niche signals during self-renewal and/or differentiation (Charalampopoulos et al., 2008; Remboutsika et al., 2011). Conclusively, on one hand, the concept of a “*rail track*” that determines the fate of cells is simple and self-explanatory. The problem is that there is not only one *cross-road*, but probably a network of *rail-roads*, where decisions are taken on the spot. Those are the fate-determining steps and their plethora, challenges our comprehension capabilities.

## Proliferation vs. regeneration

Thus, the key to developing novel and effective therapies lies with the understanding of cell growth mechanics and proliferation (Oviedo and Beane, 2009; Lambrou et al., 2013). On one hand, proliferation can be regarded as the leading effect of cancer, but whether this is the “*aetion*” (cause) (Gr. Aıτι*o*ν) or the *“aetiaton”* (causative) (Gr. Aıτıατóν) remains to be elucidated. From the beginning of the century a connection has been established between tumor growth and embryological cell growth. It was first reported by *Waddington* who mentioned and linked oncogenic mechanisms as possible regeneration mechanisms (Waddington, 1935). In particular, he stated “… *the individuation field, then, is the agent which controls the growth of the different parts in a harmonious way so that a normal individual is formed. In later life, the individuation field splits up into smaller separate fields, such as leg fields, head fields, etc. These are the agents from whom cancerous growth has escaped…*”. His work has been generally neglected in the literature, as it states that uncontrolled growth could be linked to controlled developmental growth (Slack, 2002). Furthermore, from the beginning of the century it has been evident that proliferation and regeneration are two different things. The process of regeneration, as it was observed in the embryogenesis of lower species, was derived from experimental results of transplantation in frog embryos (Needham, 1936). Another aspect of regeneration, which involves pre-existing tissue remodeling, has also been observed from the beginning of the twentieth century by *Morgan*, and was termed *morphalaxis* (Morgan, 1901). In general, a regenerative event always seeks to maintain or to re-establish the form and the function of tissue cells, a process called *morphostasis*. However, regeneration could emerge from tumor cells and as a result, tissue recovery becomes linked to cancer-related cellular anomalies (Beachy et al., 2004; Gurtner et al., 2008; Schafer and Werner, 2008; Pellettieri et al., 2010). Namely, regeneration could involve the emergence of abnormal growth, and therefore tumor growth. Based on this aspect a hypothesis is formulated. This is that the regeneration process itself, may bring under control the autonomous growth of tumor cells. The problem is that tumor emergence differs from tumor to tumor. For example, epithelial tumors and sarcomas have different mechanisms of progression and ontogenesis than other tumor types (Gibbs et al., 2005; Di Fiore et al., 2009; Lambrou et al., 2013).

As mentioned, cancer cells are able to proliferate as stem cells do. Stem cells proliferate in an orderly manner, giving rise to a part of tissue or organ. Tumor cells though are thought to undergo uncontrollable proliferation rounds. Yet, even tumor cells grow with a “*concept in mind*”. They form a pattern of growth and the “*organ*” developed is *per se* a viable organism in itself. From this concept, several motivating notions arise. Which function appears first, the ability to proliferate or the ability to regenerate? Obviously, proliferation does not always mean regeneration as in the case of cancer, yet for the tumor itself it is regeneration. If a tumor grows in an orderly manner, then for its own standards this growth is regeneration. Even the ability to grow to a sizeable structure after intense treatment is a way of regeneration, regardless of the fact that this type of growth is fatal for the implicated organism.

If we consider that stem cells exhibit two main traits, meaning the capacity to proliferate and the potential to differentiate, exactly the same traits are present in tumor cells. So what is the discriminating trait between the two cell types? The most obvious answer would be that the former grow to the benefit of the organism, while the latter to its harm. More precisely, it is suggested that tumor cells are stem cells, which are generated at the wrong place and/or time. Stem cells could be potentially the cause for tumorigenesis, when they are allocated to a tumor-instructive microenvironment. Yet, could cancer cells be in reality stem cells that start to proliferate in an uncontrollable manner creating the tumor? Is proliferation somehow always linked to regeneration? An immediate answer to this question would be no, since tumor cells do proliferate but they do not regenerate, while stem cells do regenerate when they proliferate. However, this depends on the definition of regeneration, as mentioned in the previous paragraph. Seen from the tumor “*point of view*”, a tissue with defined characteristics, which is damaged by chemotherapy, radiation or surgery, and can recover with great efficiency, can be defined as regeneration. Therefore, it is not easy to discriminate between proliferation and regeneration. It seems so that a stem cell could give rise to two different types of daughter cells. The first could lead to a healthy tissue cell, while the latter to a tumor cell. This cell fate decision between the two “*rail tracks*” lies most possibly in the microenvironment in which each cell is placed. Tumor cells were probably not *ab initio* tumor cells; they were normal cells, including both somatic differentiated cells, and stem cells, and developed into cancerous cells due to unknown etiologies.

If were to “*baptize*” each cell type with a name by citing the famous movie by *Sergio Leone*, it would be easy to attribute the term “*Good*” to a stem cell able to regenerate and produce healthy tissue in replacement of a damaged tissue; “*Bad*” to a stem cell that becomes tumorigenic and converts the proliferation process to a disease expression; and the term “*Ugly*” to the venerable stem/tumor cell, which still hasn't committed its cell fate. In other words, cells that are on a railroad track and their path is determined by numerous circumstances.

It is profound that stem cell treatment appears as a promising tool for both cancer and organ regeneration treatments. Undoubtedly, this is a fascinating area of intense research and much is yet to be learned about the biology of tumor and stem cells and their interconnecting pathways. Then, the comprehension of the delicate boundaries between proliferation and regeneration will reveal the “*mysteries”* of healing.

## References

[B1] Abelson S. Shamai Y. Berger L. Skorecki K. Tzukerman M. (2013). Niche-dependent gene expression profile of intratumoral heterogeneous ovarian cancer stem cell populations. PLoS ONE 8:e83651 10.1371/journal.pone.0083651 24358304 PMC3866276

[B2] Adegboyega P. A. Mifflin R. C. Dimari J. F. Saada J. I. Powell D. W. (2002). Immunohistochemical study of myofibroblasts in normal colonic mucosa, hyperplastic polyps, and adenomatous colorectal polyps. Arch. Pathol. Lab. Med. 126, 829–836 10.1043/0003-9985(2002)126<0829:ISOMIN>2.0.CO;2 12088453

[B3] Beachy P. A. Karhadkar S. S. Berman D. M. (2004). Tissue repair and stem cell renewal in carcinogenesis. Nature 432, 324–331 10.1038/nature03100 15549094

[B4] Calabrese C. Poppleton H. Kocak M. Hogg T. L. Fuller C. Hamner B. (2007). A perivascular niche for brain tumor stem cells. Cancer Cell 11, 69–82 10.1016/j.ccr.2006.11.020 17222791

[B5] Catalano V. Turdo A. Di Franco S. Dieli F. Todaro M. Stassi G. (2013). Tumor and its microenvironment: a synergistic interplay. Semin. Cancer Biol. 23, 522–532 10.1016/j.semcancer.2013.08.007 24012661

[B6] Charalampopoulos I. Remboutsika E. Margioris A. N. Gravanis A. (2008). Neurosteroids as modulators of neurogenesis and neuronal survival. Trends Endocrinol. Metab. 19, 300–307 10.1016/j.tem.2008.07.004 18771935

[B7] Curley M. D. Garrett L. A. Schorge J. O. Foster R. Rueda B. R. (2011). Evidence for cancer stem cells contributing to the pathogenesis of ovarian cancer. Front. Biosci. 16:368–392 10.2741/3693 21196176

[B8] Davies E. J. Marsh V. Clarke A. R. (2011). Origin and maintenance of the intestinal cancer stem cell. Mol. Carcinog. 50, 254–263 10.1002/mc.20631 21465575

[B9] De Sousa E. M. Vermeulen L. Richel D. Medema J. P. (2011). Targeting Wnt signaling in colon cancer stem cells. Clin. Cancer Res. 17, 647–653 10.1158/1078-0432.CCR-10-1204 21159886

[B10] Di Fiore R. Santulli A. Ferrante R. D. Giuliano M. De Blasio A. Messina C. (2009). Identification and expansion of human osteosarcoma-cancer-stem cells by long-term 3-aminobenzamide treatment. J. Cell. Physiol. 219, 301–313 10.1002/jcp.21667 19160414

[B11] Dvorak H. F. (1986). Tumors: wounds that do not heal. Similarities between tumor stroma generation and wound healing. N. Engl. J. Med. 315, 1650–1659 10.1056/NEJM198612253152606 3537791

[B12] Gibbs C. P. Kukekov V. G. Reith J. D. Tchigrinova O. Suslov O. N. Scott E. W. (2005). Stem-like cells in bone sarcomas: implications for tumorigenesis. Neoplasia 7, 967–976 10.1593/neo.05394 16331882 PMC1502023

[B13] Greaves M. (2010). Cancer stem cells: back to Darwin? Semin. Cancer Biol. 20, 65–70 10.1016/j.semcancer.2010.03.002 20359535

[B14] Gulhati P. Bowen K. A. Liu J. Stevens P. D. Rychahou P. G. Chen M. (2011). mTORC1 and mTORC2 regulate EMT, motility, and metastasis of colorectal cancer via RhoA and Rac1 signaling pathways. Cancer Res. 71, 3246–3256 10.1158/0008-5472.CAN-10-4058 21430067 PMC3085654

[B15] Gurtner G. C. Werner S. Barrandon Y. Longaker M. T. (2008). Wound repair and regeneration. Nature 453, 314–321 10.1038/nature07039 18480812

[B16] Jain P. Alahari S. K. (2011). Breast cancer stem cells: a new challenge for breast cancer treatment. Front. Biosci. 16, 1824–1832 10.2741/3824 21196267

[B17] Lambrou G. I. Adamaki M. Zaravinos A. (2013). Proliferation and regeneration: methodologies in cancer treatment and post-treatment tissue reconstruction, in Medical Advancements in Aging and Regenerative Technologies: Clinical Tools and Applications, ed Daskalaki A. (New York, NY: Medical Information Science Reference), 31–52

[B18] Le N. H. Franken P. Fodde R. (2008). Tumour-stroma interactions in colorectal cancer: converging on beta-catenin activation and cancer stemness. Br. J. Cancer 98, 1886–1893 10.1038/sj.bjc.6604401 18506144 PMC2441948

[B19] Morgan T. (1901). Regeneration. New York, NY: The Macmillan Company

[B20] Needham J. (1936). New advances in the chemistry and biology of organized growth: (section of pathology). Proc. R. Soc. Med. 29, 1577–1626 19990875 10.1177/003591573602901209 PMC2076269

[B21] Oishi N. Wang X. W. (2011). Novel therapeutic strategies for targeting liver cancer stem cells. Int. J. Biol. Sci. 7, 517–535 10.7150/ijbs.7.517 21552419 PMC3088875

[B22] Oviedo N. J. Beane W. S. (2009). Regeneration: the origin of cancer or a possible cure? Semin. Cell Dev. Biol. 20, 557–564 10.1016/j.semcdb.2009.04.005 19427247 PMC2706275

[B23] Pellettieri J. Fitzgerald P. Watanabe S. Mancuso J. Green D. R. Sanchez Alvarado A. (2010). Cell death and tissue remodeling in planarian regeneration. Dev. Biol. 338, 76–85 10.1016/j.ydbio.2009.09.015 19766622 PMC2835816

[B24] Remboutsika E. Elkouris M. Iulianella A. Andoniadou C. L. Poulou M. Mitsiadis T. A. (2011). Flexibility of neural stem cells. Front. Physiol. 2:16 10.3389/fphys.2011.00016 21516249 PMC3079860

[B25] Riss J. Khanna C. Koo S. Chandramouli G. V. Yang H. H. Hu Y. (2006). Cancers as wounds that do not heal: differences and similarities between renal regeneration/repair and renal cell carcinoma. Cancer Res. 66, 7216–7224 10.1158/0008-5472.CAN-06-0040 16849569

[B26] Schafer M. Werner S. (2008). Cancer as an overhealing wound: an old hypothesis revisited. Nat. Rev. Mol. Cell Biol. 9, 628–638 10.1038/nrm2455 18628784

[B27] Singh S. K. Clarke I. D. Terasaki M. Bonn V. E. Hawkins C. Squire J. (2003). Identification of a cancer stem cell in human brain tumors. Cancer Res. 63, 5821–5828 10.1038/nature03128 14522905

[B28] Singh S. K. Hawkins C. Clarke I. D. Squire J. A. Bayani J. Hide T. (2004). Identification of human brain tumour initiating cells. Nature 432, 396–401 10.1038/nature03128 15549107

[B29] Skoudy A. Hernandez-Munoz I. Navarro P. (2011). Pancreatic ductal adenocarcinoma and transcription factors: role of c-Myc. J. Gastrointest. Cancer 42, 76–84 10.1007/s12029-011-9258-0 21279552

[B30] Slack J. M. (2002). Conrad Hal Waddington: the last Renaissance biologist? Nat. Rev. Genet. 3, 889–895 10.1038/nrg933 12415319

[B31] Waddington C. (1935). Cancer and the theory of organisers. Nature 135, 6006–6608 10.1038/135606a0

[B32] Yagi H. Kitagawa Y. (2013). The role of mesenchymal stem cells in cancer development. Front. Genet. 4:261 10.3389/fgene.2013.00261 24348516 PMC3842093

[B33] Zheng T. Wang J. Jiang H. Liu L. (2011). Hippo signaling in oval cells and hepatocarcinogenesis. Cancer Lett. 302, 91–99 10.1016/j.canlet.2010.12.008 21247686

